# Understanding the response to endurance exercise using a systems biology approach: combining blood metabolomics, transcriptomics and miRNomics in horses

**DOI:** 10.1186/s12864-017-3571-3

**Published:** 2017-02-17

**Authors:** Núria Mach, Yuliaxis Ramayo-Caldas, Allison Clark, Marco Moroldo, Céline Robert, Eric Barrey, Jesús Maria López, Laurence Le Moyec

**Affiliations:** 1 Animal Genetics and Integrative Biology unit (GABI), INRA, AgroParisTech, Université Paris-Saclay, 78350 Jouy-en-Josas, France; 2 Health Science Department, Open University of Catalonia (UOC), Barcelona, Spain; 3 Paris-Est University, National Veterinary School of Alfort, Maisons-Alfort, France; 4 Integrative Biology of Exercise Adaptations unit, UBIAE, EA7362, Evry Val d’Essone University, Evry, France

**Keywords:** Endurance exercise, Horse, Metabolome, miRNome, Multiple factor analysis, Regulome, Transcriptome, Systems biology

## Abstract

**Background:**

Endurance exercise in horses requires adaptive processes involving physiological, biochemical, and cognitive-behavioral responses in an attempt to regain homeostasis. We hypothesized that the identification of the relationships between blood metabolome, transcriptome, and miRNome during endurance exercise in horses could provide significant insights into the molecular response to endurance exercise.

For this reason, the serum metabolome and whole-blood transcriptome and miRNome data were obtained from ten horses before and after a 160 km endurance competition.

**Results:**

We obtained a global regulatory network based on 11 unique metabolites, 263 metabolic genes and 5 miRNAs whose expression was significantly altered at T1 (post- endurance competition) relative to T0 (baseline, pre-endurance competition). This network provided new insights into the cross talk between the distinct molecular pathways (e.g. energy and oxygen sensing, oxidative stress, and inflammation) that were not detectable when analyzing single metabolites or transcripts alone. Single metabolites and transcripts were carrying out multiple roles and thus sharing several biochemical pathways.

Using a regulatory impact factor metric analysis, this regulatory network was further confirmed at the transcription factor and miRNA levels.

In an extended cohort of 31 independent animals, multiple factor analysis confirmed the strong associations between lactate, methylene derivatives, miR-21-5p, miR-16-5p, let-7 family and genes that coded proteins involved in metabolic reactions primarily related to energy, ubiquitin proteasome and lipopolysaccharide immune responses after the endurance competition. Multiple factor analysis also identified potential biomarkers at T0 for an increased likelihood for failure to finish an endurance competition.

**Conclusions:**

To the best of our knowledge, the present study is the first to provide a comprehensive and integrated overview of the metabolome, transcriptome, and miRNome co-regulatory networks that may have a key role in regulating the metabolic and immune response to endurance exercise in horses.

**Electronic supplementary material:**

The online version of this article (doi:10.1186/s12864-017-3571-3) contains supplementary material, which is available to authorized users.

## Background

Endurance exercise can be defined as cardiovascular exercise —such as running, cross-country skiing, cycling, aerobic exercise or swimming— that is performed for an extended period of time [1, 2]. Endurance athletes expose their bodies to extreme physiological conditions that disrupt homeostasis [2, 3]. The main adaptations to endurance exercise include changes in neuromuscular and contractile functions in muscles [4], correction of electrolyte imbalance [5], decreased glycogen storage [6], increased mitochondrial biogenesis in muscle tissue [7], body temperature regulation, oxidative stress [7], increased intestinal permeability and hypoperfusion, muscle damage, systemic inflammation and immune responses [8]. Additionally, the physical demands during intense exercise can trigger a stress response activating the sympatho-adreno-medullary and hypothalamus-pituitary-adrenal (HPA) axes, which results in the release of corticotrophin-releasing hormone, arginine vasopressin, adrenocorticotropic hormones, glucocorticoids and catecholamines into the circulatory system (reviewed by Clark and Mach [3]).

Horses serve as an optimal in vivo model for characterizing the response to endurance exercise due to their natural aptitude for athletic performance and the homogeneity of their genetic and environmental backgrounds [9]. As described by Capomaccio et al. [9], the effort that an equine athlete exerts during an endurance competition is comparable to that of a human marathoner or ultra marathoner.

Over the past decade, the transcriptional and translational mechanisms of gene regulation that control the responses to endurance exercise have been studied by many authors [8, 10–16]. However, gene and miRNA expression data might only indicate the potential physiological effects because many pathway feedback mechanisms are simply not reflected in gene expression changes [17]. For this reason, metabolomics, which focuses on the final “omic” layer of a biological system, has emerged as a more integrative approach towards the understanding of the biological functions of an organism [18]. It has been recently shown that proton nuclear magnetic resonance (^1^H NMR)-based metabolomic analysis of horse [19, 20] and human [21] serum employing unsupervised statistical methods enabled the detection of certain classes of metabolites such as lactate, amino acids, lipids, nucleic acids, and secondary metabolites whose levels had been modified in response to endurance exercise. Metabolites, such as those previously mentioned, are the result of a continuum of many integrated enzymatic and non-enzymatic steps, which include numerous intermediaries that are regulated by the host genome, epigenome, metagenome, food and drink consumption, drug use and exposure to pollutants.

To date, most studies in the field of metabolomics have failed to fully explain all of the alterations that occur during the metabolic regulatory processes, tissue lesions or organ dysfunctions that athletes face when coping with stress responses during endurance exercise [18, 20, 22–25]. The need for a deeper understanding of how these regulatory metabolic networks function in response to endurance exercise has led to increased efforts to model multiple “omic” dimensions simultaneously. Therefore, we performed an integrated analysis of the blood metabolome, transcriptome and miRNome in horse athletes that participated in an endurance competition. We hypothesized that the identification of the relationships between blood metabolome, transcriptome and miRNome, which are specifically regulated by endurance exercise in horses, could (i) characterize the complex interplay between serum metabolome and whole-blood transcriptome and miRNome data; and (ii) reveal unique biomarkers of reactions to stress during endurance exercise.

## Results

The morphological and physiological parameters of the 41 equine athletes used in the study are summarized in Additional file 1, whereas the biochemical parameters obtained from blood samples collected after the endurance competition are represented in the Additional file 2. All horses showed above-average total bilirubin, creatine kinase, aspartate transaminase, and serum amyloid A concentrations, reflecting hemolysis and muscular membrane permeability or inflammation.

### Endurance competition affected the expression of a large number of genes, miRNAs and metabolites

^1^H NMR spectra together with custom equine mRNA and miRNA microarrays were used on an experimental set of ten horses to study the effects that endurance exercise has on the blood metabolome, transcriptome and miRNome. Before delving into multi-layered data sets integration for the analysis of the regulatory network, we assessed the quality and significance of each “omic” layer individually.

**Fig. 1:**
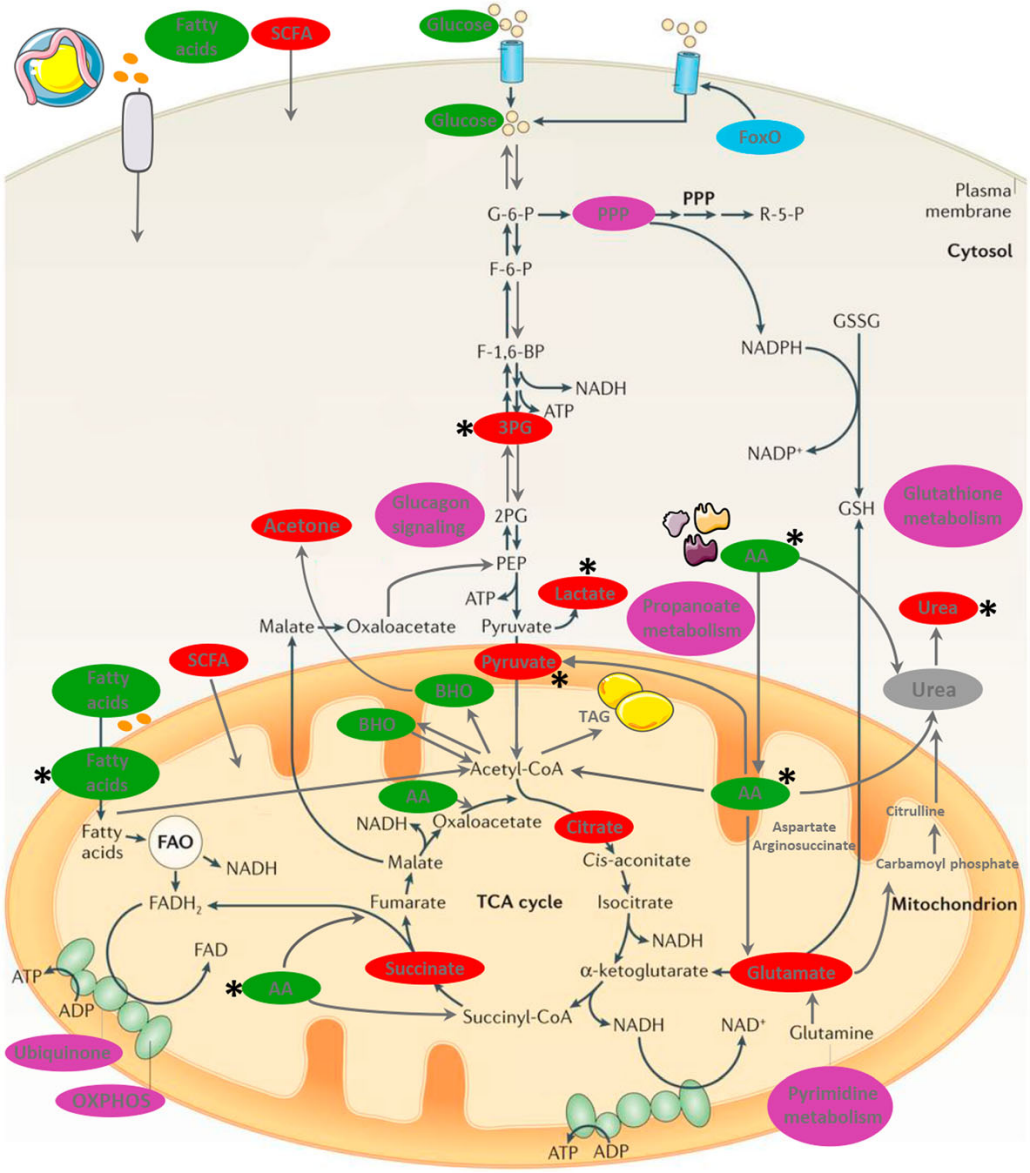
Metabolic regulation in the cell after endurance exercise. Endurance exercise increased the production of pyruvate from anaerobic glycolysis to compensate for ATP production. On one hand, pyruvate was converted into lactate. On the other hand, pyruvate entered in the TCA cycle to produce NADH and semiquinone. The OXPHOS of fatty acids increased, while amino acids were deaminated to fuel the TCA cycle during the endurance competition, which increased the production of ammonia. Because glucose levels become too low during endurance exercise, the keto acid oxaloacetate was preferentially utilized in the process of gluconeogenesis, instead of reacting with acetyl CoA, and diverted to ketone body formation (e.g. acetone). In the figure, the node color intensity indicates the associated expression level: *red* = over-expression at T1 and *green* = under-expression at T1. G-6-P, glucose 6-phosphate; F-6-P, fructose 6-phosphate; F-1, 6-BP, fructose-1,6- bisphosphate; GSH, reduced glutathione; GSSG, oxidized glutathione; PEP, phosphoenolpyruvate; R-5-P, ribose-5-phosphate. *denotes statistical significance at 0.05 level. Figure adapted from Kruiswijk et al. [62] with permission from Nature publishing group (License number: 3902041297601)

A total of 54 metabolic peaks were identified, including several amino acids, energy metabolism-related metabolites, saccharides, and organic osmolytes in the plasma (Fig. 1). Among the 54 metabolic peaks identified, we observed two unassigned compounds at 3.72–3.76 and 3.85–3.88 ppm, respectively (Additional file 3).

The effect exercise had on the serum metabolome was described by multivariate statistical methods. On one hand, the principle component analysis (PCA) significantly discriminated pre and post-endurance competition samples (Monte Carlo test; *p* < 0.05; Additional file 4: Figure S1A). On the other hand, the orthogonal projections to latent structures (OPLS) analysis revealed that a total of 49 metabolites resonances contributed in the discrimination of the pre and post-endurance competition samples (Additional file 4: Figure S1C). Lactate, followed by methylene, N-acetyl moieties, proline, glucose and phosphocholine presented the highest contribution to class separation (Additional file 4: Figure S1C). More precisely, loadings examined on the pre-component basis as line plot emphasized that post-competition samples were characterized by higher excursion of lactate, glycerol, creatine, urea, and aromatic amino acids such as tyrosine, along with negative excursion of methylene, N-acetyl moieties, methylene esters, glucose, and phosphocholine (Additional file 4: Figure S1C).

To ensure the accuracy of the OPLS model (*R* ^2^ = 0.88 and Q^2^ = 0.92), permutations testing and cross validation were used for internal validation of the OPLS model. The explained variance, predictive capability and out-of-bag error of OPLS model remained higher (*R* ^2^ = 0.57 ± 4.07; Q^2^ = 0.94 ± 0.03 and root mean square error of prediction (RMSEP) = 0.30 ± 0.09) than those of the 1,000-permutated models (*R* ^2^ = 0.53 ± 7.04; Q^2^ = 0.16 ± 0.48 and RMSEP = 0.65 ± 0.16) to discriminate pre- and post-endurance competition samples (*p* < 0.001).

The relationship between metabolites and the functional map of the metabolites affected by the endurance exercise are provided in the Additional file 5: Figure S2 and Additional file 6: Figure S3, respectively. Among the most enriched pathways are those involved in glycerolipid metabolism, D-glutamine and D-glutamate metabolism, pyruvate metabolism, tricarboxylic acid (TCA) cycle and amino acids metabolism (Additional file 6: Figure S3).

Afterwards, we focused on the identification of genes and miRNAs whose expression was significantly altered at T1 (post-endurance competition) relative to T0 (baseline, pre- endurance competition), with an adjusted *p*-value < 0.05 after implementing Benjamini and Hochberg correction for multiple testing (Additional file 7). The application of this threshold led to the identification of 7,678 differentially expressed genes (DEGs), 3,848 of which were over-expressed and 3,830 of which were under-expressed at T1 (relative to T0). We identified 107 miRNAs differentially expressed (DEmiRNAs) when comparing pre- and post- endurance competition samples (Additional file 8). Along with orthologous human-equine DEmiRNAs, we detected 7 equine-specific DEmiRNAs.

### A regulatory network of 11 metabolites, 263 genes, and 5 miRNAs has a key role in controlling the adaptation to endurance exercise

The 49 metabolite resonances that significantly discriminated between pre and post- endurance competition samples, together with the 7,678 DEGs and 107 DEmiRNAs were used to perform a multi-step approach and identify the dynamically co-regulated relationship among metabolites, genes and miRNAs that could not be detected when analyzing only single metabolites or transcripts alone (Additional file 9: Figure S4).

We performed two different complementary statistical approaches (detailed in the Methods section) to identify enriched metabolites with significantly more predictive metabolic genes among the DEGs than among other genes in the transcriptome. The enzyme-encoding genes whose protein products participate in the metabolic reaction of a given metabolite are referred to here as metabolic genes. Next, for each of the metabolic genes, we used the multiMiR package in R to build a comprehensive list of all the putative DEmiRNA regulators.

**Fig. 2:**
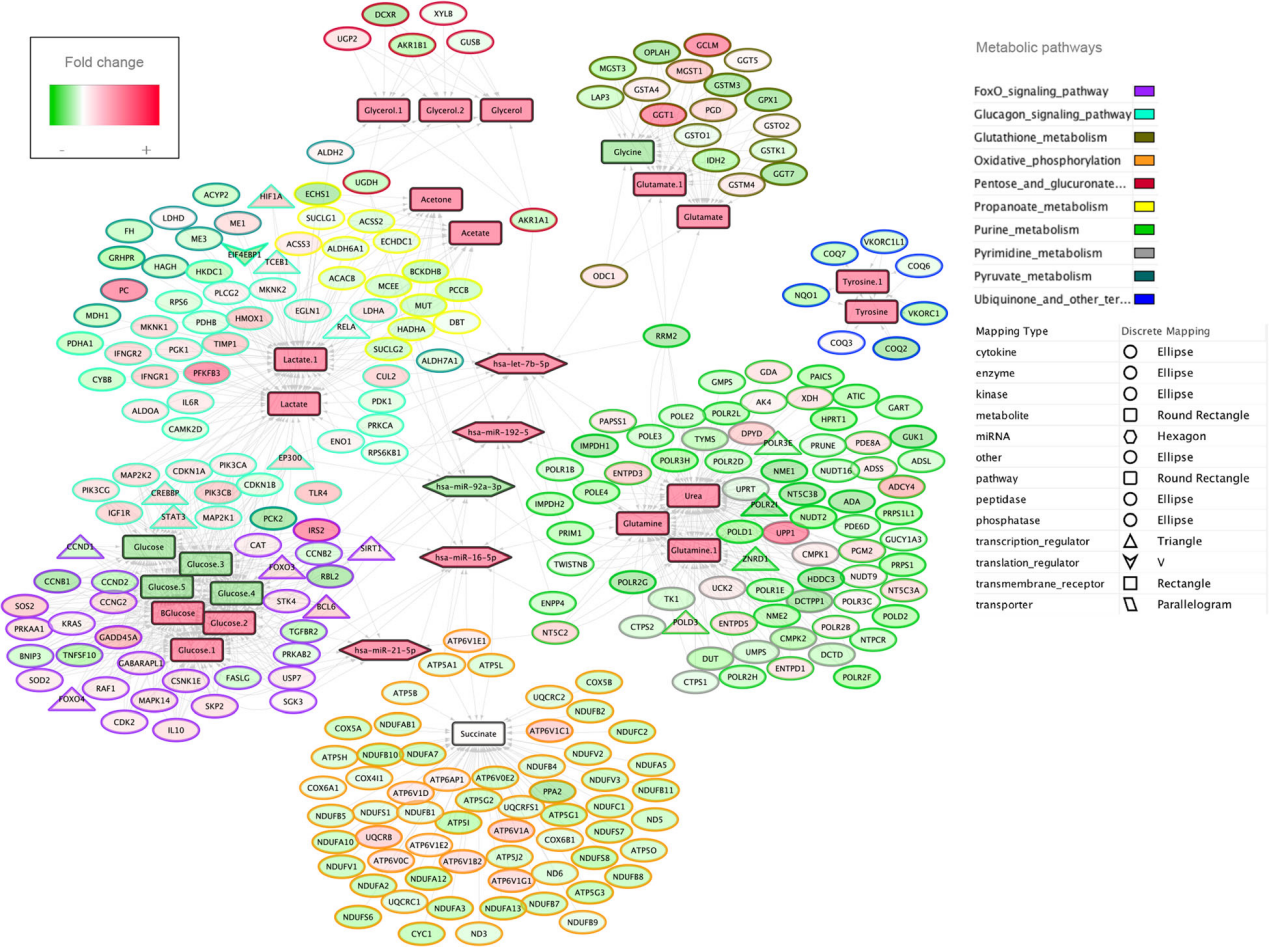
Regulatory network linking metabolites, metabolic genes and miRNAs. We identified a total of 11 unique metabolites, which were associated with a total of 263 unique metabolic target genes and 5 miRNAs. The network is displayed graphically as nodes (genes, TFs and miRNAs) and edges (biological relationships). The node color intensity indicates the expression level of the association: *red* = over-expression at T1 and *green* = under-expression at T1. The node shape indicates whether the node is a TF (*triangles*), a miRNA (*hexagon*), a metabolite (*round rectangle*), membrane receptor (*rectangle*), transporter (parallelogram), or other type of genes (*ellipses*)

In particular, we found a total of 11 differentially expressed metabolites (Additional file 10) that were putatively regulated by a total of 263 differentially expressed genes. Interestingly, we discovered that the spatiotemporal expression of those 263 metabolic genes could be extensively regulated at the transcription and post-transcription level through a total of 79 DEmiRNAs. For instance, let-7b-5p, miR-16-5p, miR-21-5p, miR-92a-3p, and miR-192-5p regulated more than ten metabolic genes each (Fig. 2).

Ultimately, we considered a total of 11 enriched metabolites correlated with the expression of 263 metabolic genes and 5 miRNAs during exercise to create the regulatory network (Fig. 2). This regulatory network underlined several well-known biochemical interactions, i.e., the relationships between the decreased glucose concentration after endurance exercise and the metabolic genes whose proteins participate in the Forkhead box protein O (FoxO) and glucagon signaling pathways to induce gluconeogenesis, and the relationship between lactate and metabolic genes encoding proteins related to glucagon signaling and propanoate metabolism (Fig. 2). Another well-known biochemical interaction was found between succinate and the expression of multiple components of the mitochondrial oxidative phosphorylation (OXPHOS) system, including multiple subunits of ATP synthase (seven Fo subunits and the *ATP5A1, ATP5B* and *ATP5O* subunit of F1), NADH: ubiquinone oxidoreductase core subunits (*ND3, ND5, ND6*; complex I) and cytochrome oxidase subunits (*COX4I1, COX5A, COX5B, COX6A1, COX6B1*). As expected, the regulatory network also highlighted the interaction between glycerol and metabolic genes that encode enzymes participating in the pentose and glucuronate pathways, as well as acetate and many downstream genes of propanoate metabolism, which included activated protein kinase (AMPK) activators such as acetyl-CoA carboxylase (*ACACB*) and acyl-CoA synthetase short-chain family (*ACSS2, ACSS3*).

These well-known biochemical interactions were mainly regulated by miR-16-5p, miR-21-5p, miR-92a-3p, miR-192-5p and let-7b-5p within the regulatory network.

Remarkably, the “omic” integration of molecular data in our study predicted some functional links between metabolites and the transcriptome that could not be characterized by analyzing separately any of the three individual data sets. One example is the functional enrichment of glutamate through metabolic genes belonging to the glutathione metabolism, which regulates the reactive oxygen species (ROS) and nitrogen oxide species (RONS) substances (e.g. the glutathione S-transferase (*GST*) gene family, GST-kappa 1 (*GSTK1*), and two microsomal GSTs (*MGST1* and *MGST3*). In line with the exercise-induced oxidative stress, we observed a significant interaction between tyrosine and metabolic genes that affected the ubiquinone endogenous antioxidant levels. Another example of the importance of “omic” integration was represented by the interaction between urea and genes involved in the catabolism of purine and pyrimidine nucleotides from ATP to uric acid, in which free radicals are produced and cause muscle cell damage [26]. Lastly, a significant interaction that could not be characterized by analyzing separately any of the three individual data sets was the link between lactate and the activation of the hypoxia-inducible factor A (*HIF1A*) transcription factor, as well as lactate genes associated with the release of lipopolysaccharides (LPS)-induced pro-inflammatory cytokines, including toll-like receptor 4 (*TLR4*), followed by the activation of various immune-related transcription factors (TFs) such as signal transducer and activator of transcription 3 (*STAT3*) and the nuclear factor kappa beta (*NFkβ-RelA*).

**Fig. 3:**
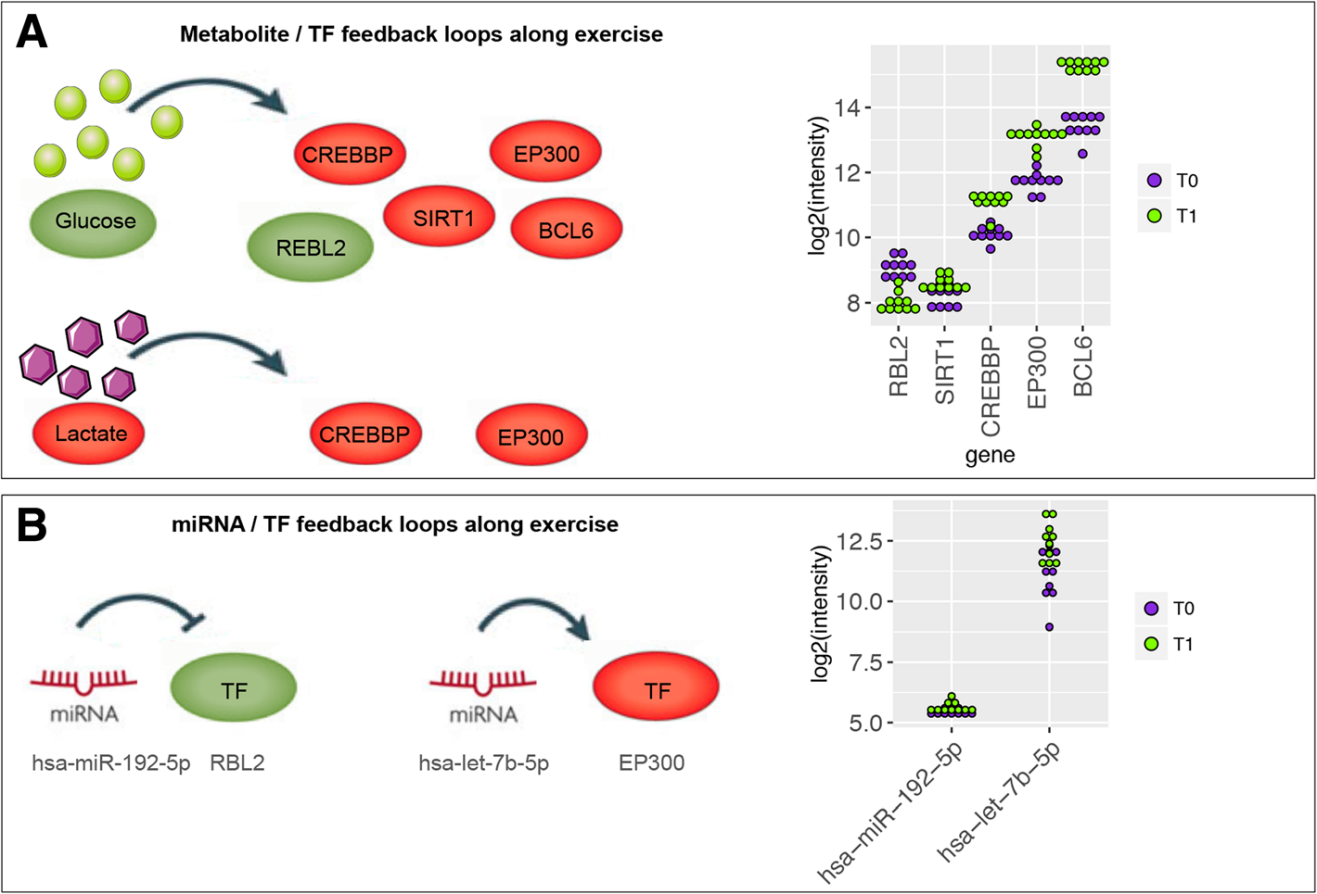
Activators and repressors of the regulatory network. **a** The regulatory network was driven by different transcription factors able to up- or down regulate the glucose and lactate concentration (*EP300, CREBBP, SIRT1, BCL6,* and *REBL2*). **b** The *EP300* and *SIRT1* transcription factors could be regulated by the expression of let-7b-5p and miR-223-3p, respectively, and *RBL2* might be inhibited by miR-192-5p. In the figure, the node color intensity indicates the associated expression level: *red* = over-expression at T1 and *green* = under-expression at T1

Moreover, we found that the regulatory network based on the 263 metabolic genes included TFs such as retinoblastoma-Like 2 *(RBL2),* followed by the cooperatively transcriptional cofactors sirtuin 1 (*SIRT1),* E1A binding protein P300 (*EP300*), CREB Binding Protein (*CREBBP*), and B-cell lymphoma 6 protein (*BCL6*; Fig. 3a). Additionally, results suggested that these TFs might (i) regulate the spatiotemporal concentration patterns of glucose and lactate (Fig. 3a); and (ii) drive or repress the expression of metabolic genes and miRNAs in a feed-forward and feedback manner [27] (Fig. 3b).

### The regulatory network is driven by both transcription factors and miRNAs

As suggested above, both TFs and miRNAs might regulate endurance exercise response. In order to ensure the accuracy of TFs and miRNAs regulation within the regulatory network, we decided to use the regulatory impact factor (RIF) algorithm [28, 29]. Our aim was to identify (i) putative causal regulators, and (ii) the rewired transcriptional circuits through which the TFs and miRNAs exert their regulatory impact on the transcriptome following exercise.

Among the most enriched TFs and miRNAs, we confirmed *CREBBP* (RIF2 = -2.51)*, EP300* (RIF2 = -1.55)*, RBL2* (RIF1 = 1.65), *SIRT1* (RIF1 = 1.63), *BCL6* (RIF2 = 1.48) and let-7b-5p (RIF1 = 1.51; Fig. 2). Details on the top regulators with greatest scores are fully listed in the Additional file 11.

### The relationship between blood metabolome, transcriptome and miRNome that occurs during endurance exercise could be reproduced in an independent cohort of 31 horses

As a final analysis step, multiple factor analysis (MFA) was applied to an independent cohort of 31 horses to further emphasize the reliability of cross-layer “omic” analysis to predict or explain the exercise response adaptations and importantly, to confirm the interactions within our regulatory network. This independent cohort included 13 horses sampled only at T0, and 18 other horses sampled only after completing the endurance competition (T1).

**Fig. 4:**
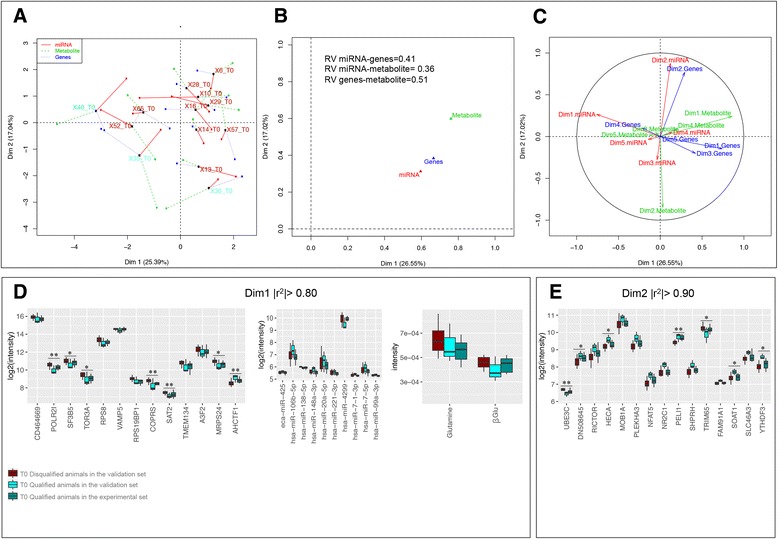
Multiple factor analysis projection plot at T0 in an independent cohort of 13 animals. **a** The different “omics” data sets are connected by lines where the length of the line is proportional to the divergence between the data from a same animal. Lines are joined by a common point, representing the reference structure, which maximizes covariance derived from the MFA synthetic analysis; **b** Summarization of the concordance between “omics” data set on the space; **c** “Omics” data sets are shown in the different dimensions while showing the contribution of each eigenvalue to the total variance; **d** Boxplot of the expression of the molecules having high weights (|r^2^| > 0.80) on the PC1 of MFA; **e** Boxplot of the expression of genes having high weights (|r^2^| > 0.90) on the PC2 of MFA. In all cases, animals that finished the endurance competition in the validation set were colored in cyan color, whereas animals in the validation set that were disqualified during the endurance competition were colored in dark red color. Additionally, to ensure that expression levels of the animals in the validation set that finished the endurance competition (*n* = 3) were similar to those of the experimental set that finished the endurance competition (*n* = 10), for each molecule, expression levels of animals from experimental set (*dark green color*) were also plotted. *, ** denote statistical significance at the 0.10 and 0.05 level respectively

At T0 (basal time), the MFA superimposed plot of each type of data and its barycenter showed particularly high levels of variability in all “omic” layers (Fig. 4a). However, the MFA projection plot of the partial representations for each “omic” layer (metabolome, transcriptome and miRNome) onto PC1 was tightly clustered (RV-coefficient = 0.51; Fig. 4b). The two metabolites showing the highest correlation (|r^2^| > 0.80) on the first axis were glutamine and glucose, whereas the genes most strongly correlating with PC1 (|r^2^| > 0.80) were RNA polymerase 21-kDA subunit *(POLR2I),* torsin 3A *(TOR3A),* vesicle-associated membrane protein 5 (*VAMP5*), histone binding protein 5 (*COPR5)*, N1-acetyltransferase family member 2 (*SAT2*), AT-hook containing transcription factor 1(*AHCTF1*) and various ribosomal proteins (Fig. 4d). The eca-miR-425, miR-106b-5p, miR-138-5p, miR-148a, and miR-20a-5p were also highly linked to the first principal component (PC) (|r^2^| > 0.80; Fig. 4d). Genes such as E3 ligases (*UBE3C*) and tripartite motif containing 65 (*TRIM65*) presented high correlations with PC2 (|r^2^| > 0.90; Fig. 4e). The detailed weights of each metabolite, metabolic genes and miRNA are provided in Additional file 12.

Although the size of the cohort used at T0 was relatively small, the MFA analysis revealed that the partial representation of the mean individuals for each “omic” layer was projected together according to their capacity to finish the endurance competition (Fig. 4a). Thus, the molecular profiles at basal time of the animals that did not finish the endurance competition were projected close to each other in the space (Fig. 4a). In light of these findings, we wanted to understand whether the molecules detected at basal time could be considered as predictive biomarkers for the elimination during the endurance competition. Although cause and effect are usually difficult to decipher, we compared basal profiles of molecules that showed high correlation values (|r^2^| > 0.80) on the two first dimensions of the MFA with animals that finished the endurance competition and animals that were eliminated during the endurance competition because of lameness or metabolic problems. We discovered that the *POLR2I, COPR5, SAT2* and *UBE3C* genes were clearly up regulated (corrected *p* < 0.05) at basal time in animals that were eliminated during the endurance competition, whereas *TOR3A* and *TRIM65* tended to be up regulated (corrected *p* < 0.10) at basal time. They also presented numerically higher glutamine and glucose concentrations at basal time (Fig. 4d).

**Fig. 5:**
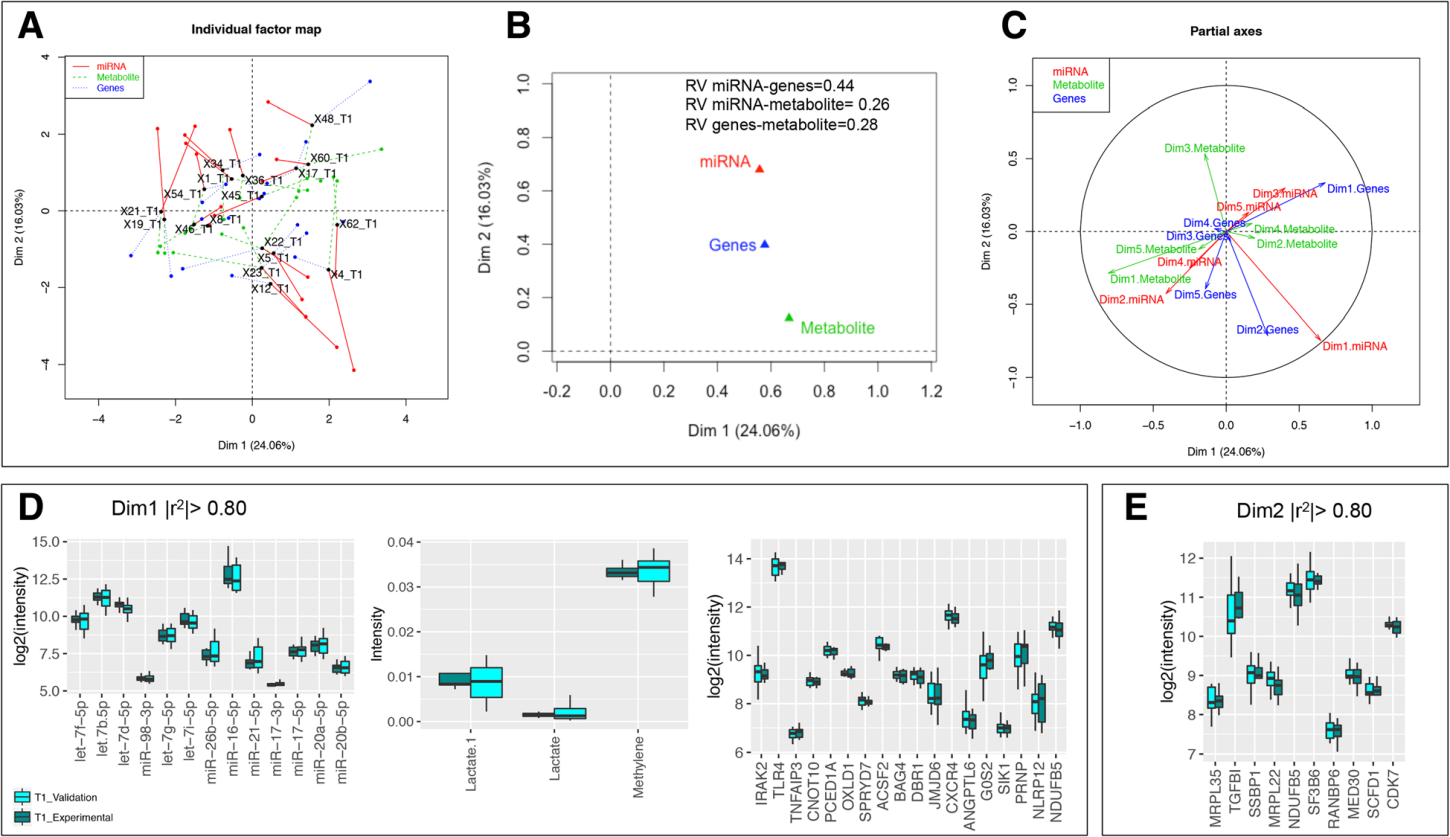
Multiple factor analysis projection plot at T1 in an independent cohort of 31 animals. **a** The first two axes of MFA represent metabolome, transcriptome, and miRNome data sets at T1. Different shapes and colors represent the respective “omic” data sets, which are connected by lines where the length of the line is proportional to the divergence between the data from a same animal. Lines are joined by a common point, representing the reference structure, which maximizes covariance derived from the MFA synthetic analysis. **b** Summarization of the concordance between “omic” data sets on the space. **c** Data sets are shown in the different dimensions while showing the contribution of each eigenvalue to the total variance; **d** Boxplot of the expression of the molecules having high weights (|r^2^| > 0.80) on the PC1 of MFA; **e** Boxplot of the expression of genes having high weights (|r^2^| > 0.80) on the PC2 of MFA. In all cases, to ensure that expression levels of the animals in the validation set confirmed those of the experimental set, for each molecule, expression levels of animals from the validation set (*cyan color*) and experimental set (*dark green color*) are plotted

The combined analysis of metabolome, transcriptome and miRNome on post-exercise samples then highlighted an interesting co-structure between data sets (Fig. 5). Metabolome profile accounted for the highest variance on PC1, whereas the miRNome data were projected on the positive end of the PC2 (Fig. 5b). Metabolites such as lactate and fatty acid methylene moieties presented high correlation values |r^2^| > 0.80 with PC1, together with let-7 family, miR-16-5p, miR-17, miR-20ab-5p, miR-21-5p,miR-26b-5p and miR-98-3p (Fig. 5d). The LPS-induced immune markers, specifically *TLR4*, TNF alpha induced protein 3 (*TNFAIP3*) and interleukin-1 receptor-associated kinase-like 2 (*IRAK2*), were identified among the strongest variables of the PC1 (Fig. 5d), along with the increase in expression of genes related to mitochondrial metabolism (e.g. NADH dehydrogenase (ubiquinone) 1 beta sub-complex 5; *NDUFB5*).

Additional file 13 shows an expanded list of the genes related to LPS-induced immune responses with its MFA impact factor at T1. Biologically interesting genes with high correlation values on the PC2 were the mitochondrial ribosomal protein L35 and L22, respectively (*MRPL35*, *MRPL22*), and mitochondrial single-stranded DNA-binding protein (*SSBP1*). The top molecules with greatest weights on PC1 and PC2 at T1 are listed in the Additional file 13.

## Discussion

The level of exercise performed by horses during an endurance competition is similar to that of a human ultra marathon runner (from 50 km to 160.934 km) [30] or to a lesser extent a marathon runner (42.195 km) [9]. Endurance horses run 80–160 km endurance competitions that require energy expenditure and cause physical stress that the body must adapt to through coordinated metabolic, immune and hormonal responses in order to maintain homeostasis. Our previous work on plasma metabolome profiling mainly demonstrated that adaptation to endurance exercise in horses simultaneously involved lipid, protein catabolism and glycoprotein pathways [19]. Our previous study on blood transcriptome and miRNome response to endurance exercise in horses mainly reflected immune system processes through white blood cells as well as regulatory processes involved in various pathways such as glucose metabolism, fatty acid oxidation and mitochondrion biogenesis [8]. Until this current study, our prior work had been limited to single levels of biological information, therefore neglecting the global network structure and cross talk that occurs across multiple layers of molecular organization.

No systematic exploration of information flow between the different biological “omic” layers had been carried out in athletes. Therefore, we decided to explore the metabolic plasticity in response to endurance exercise by simultaneously combining plasma metabolite changes together with the blood transcriptome and miRNome from the same individuals. A similar study in humans had already described that the concurrent analysis of cross-sectional multi “omic” data from the same individuals could be a powerful tool to identify the underlying molecular mechanisms that occur on a physiological scale such as fasting in elderly (68.82 ± 4.31 years) [31].

Integrating the results from circulating plasma metabolites, transcriptome and miRNome derived from the blood of ten endurance horses before and after an endurance competition, allowed us to reveal biologically reasonable relationships after the endurance competition, namely: (i) reduction of glucose concentration and inhibition of FoxO signaling, which up-regulate genes involved in gluconeogenesis, lipid metabolism and ketone body production and utilization, and activation of miR-21-5p expression [32]; (ii) increased OXPHOS to compensate for ATP production; and (iii) increased concentration of acetate, which subsequently induced AMPK [33, 34].

Additionally, we found several associations within our regulatory network, which to the best of our knowledge have not been fully described before. For example, our study showed that glutamate was enriched by co-expressed genes that regulate the control and removal of ROS and RONS molecules, such as the *GST* family, *GSTK1*, and two microsomal GSTs, suggesting that under physical stress, glutamate might be used for the synthesis of glutathione, a major endogenous antioxidant in mammalian cells. This is in agreement with the observed positive correlation between tyrosine (another effective antioxidant in biological fluids [26]) and the expression of genes associated with ubiquinone synthesis, which are redox active and essential lipophilic electron carriers of the mitochondrial electron transport chain involved in the Q-cycle [26]. Collectively, the results suggest that an elevated respiration rate during endurance exercise may have led to the generation of more ROS than the antioxidant systems can scavenge, and that the increase in muscle injury (confirmed by high levels of plasma aspartate transaminase and serum amyloid A) also reflected an increase in the generation of the ROS to levels greater than the antioxidants can handle.

**Fig. 6:**
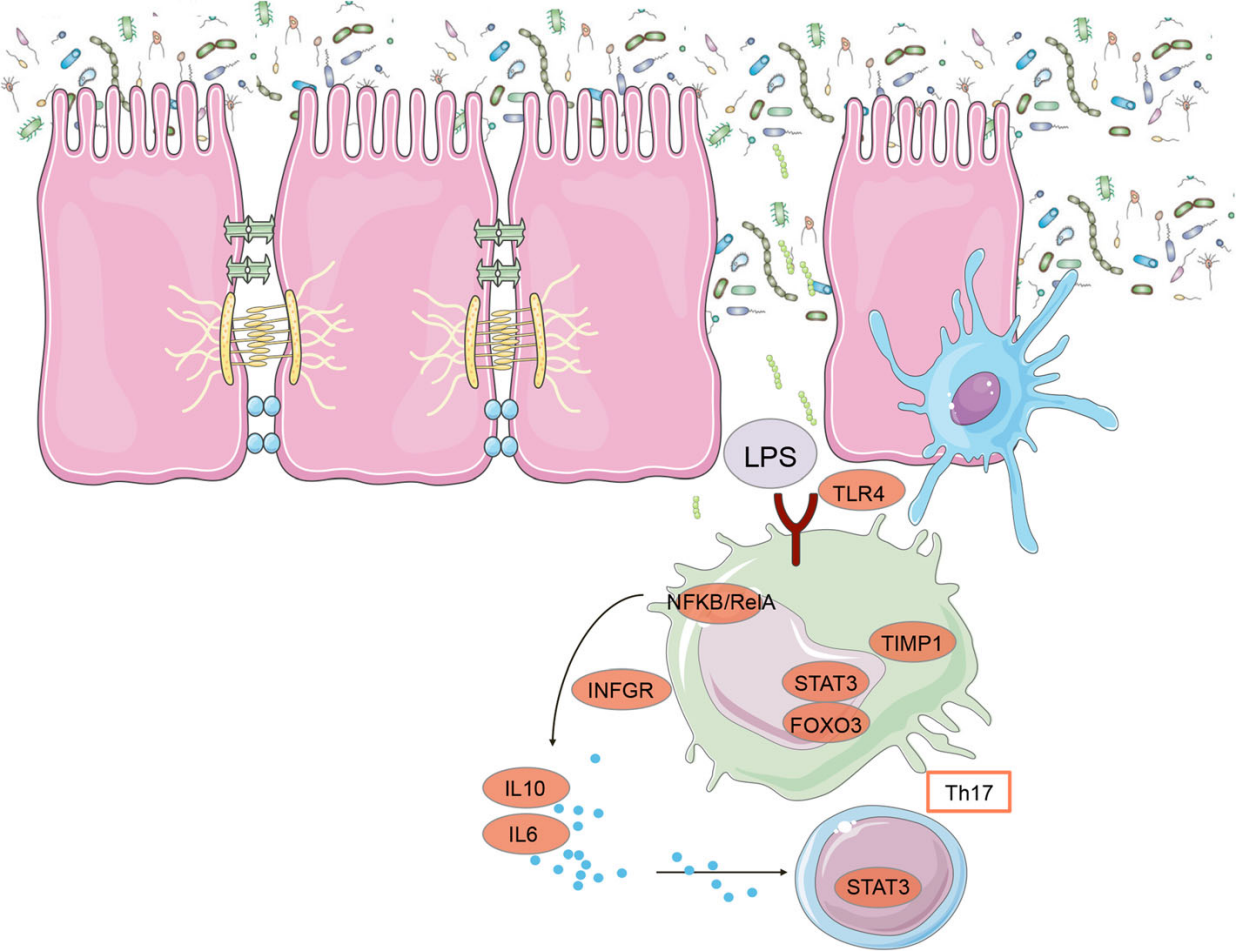
A model for increased intestinal permeability after exercise based on coordinated metabolite and gene expression after the endurance competition. Intestinal homeostasis involves the coordinated actions of epithelial, innate and adaptive immune cells. Our data showed an increase in *TLR4*, which probably stimulated the release of pro-inflammatory cytokines such as interferon alpha and gamma (IFNα, INFγ), or interleukin (IL) 6 (all of them up-regulated after exercise) and sensed the translocated LPS. We also observed an increase of *FoxO3*, which regulates IL10 expression during a typical LPS immune response, as well as *STAT3, NfkB-RelA*, and interferon-gamma receptor (*IFNGR-1)*. This also correlated with the observed increase in blood serum amyloid protein (SAA) in the post- endurance competition samples, an inflammatory marker. The node color indicates whether the node is a gene (*orange*), or a metabolite (*violet*). This figure was produced using Servier Medical Art, available from www.servier.com/Powerpoint-image-bank

The presumably persisting oxidative stress and cell damage after competition was further confirmed by two different relationships within our regulatory network: (i) the relationship between lactate and genes that were likely involved in LPS-induced pro-inflammatory pathway (Fig. 6), and (ii) the relationship between urea and genes involved in energy-purine and pyrimidine catabolism, which could result in the accumulation of uric acid and toxic oxygen free radicals in the cells [26]. Of note, miR-92a-3p and miR-192-5p, which share the same seed sequence, were related to oxidative stress genes. As previously reported, the expression of miR-92a is regulated negatively by oxidative stress [35].

To further emphasize the reliability of cross-layer “omic” analysis to predict or explain the endurance response adaptations and importantly, and to confirm the molecules within our regulatory network and their interactions, we applied the MFA approach, which can infer direct relationships between variables within a set of repeated observations in the absence of a priori knowledge. Therefore, we used an independent cohort of 31 horses and we considered horses at T0 and horses at T1 separately. For each scenario (T0 or T1), MFA was used to model interactions between the metabolome, the transcriptome and the miRNome. At basal time, the MFA approach gave insights about the reasons why a large number of horses analyzed in our independent cohort at T0 failed to finish the competition (*n* = 10). This indicated potential future candidate biomarkers that could predict the increased likelihood for failure to finish an endurance competition. Our MFA analysis suggested that the horses eliminated during the endurance competition already presented metabolic and inflammatory issues prior to the endurance competition possibly due to poor training preparation and feeding management, uncomfortable transport or other physiological reasons (e.g. increased intestinal permeability, lameness, muscle injury). Thus, the exercise-induced stress experienced during the 160 km competition made it more difficult for them to control their energy and inflammation levels. For instance, proteins (represented by their respective transcripts) related to LPS-induced immune responses (e.g. TOR3A [36], UBE3C [37]), and inflammation in the skeletal muscle tissue (e.g. COPR5 [38]) were higher in animals that were eliminated during the endurance competition compared to horses that successfully finished the competition. Additionally, horses that were eliminated during the endurance competition presented higher glutamine and *SAT2* expression, which is the transporter responsible for glutamine uptake in the neuronal compartment making them important components of the glutamate/GABA-glutamine cycle [39]. A study by Keller et al. [40] using ^1^H NMR metabolomic analysis of blood from horses with laminitis also showed increased glutamine levels, further pointing to metabolic issues as the underlying cause for elimination in these horses. Overall, these results suggest that analyzing the expression of metabolic and transcript signaling before starting the endurance competition could be a useful tool to predict elimination during the endurance competition at basal time, though larger data sets studies aimed at investigating the predictive value of these biomarker candidates are needed.

When exploring the associations between biological processes across different biological layers in the post- endurance competition samples of our independent cohort, MFA highlighted the significant role that genomic factors play a LPS-induced immune response, together with mitochondrial *SSPB1*, which protects cells from proteotoxis stress by increasing stress-induced heat-shock factor 1 (*HSF1*) transcriptional activity [41]. We therefore confirm our hypothesis that an excessive release of stress hormones induced by physical stress during the endurance competition together with increased body oxygen uptake, lead to the generation of ROS and RONS in working muscles and in the tissues that undergo ischemia and hypoperfusion [26]. These processes are associated with cell damage and LPS translocation outside of the gastrointestinal tract, which in turn triggers immune and inflammatory responses often resulting in increased intestinal permeability and ROS [3]. In humans, depending on the type of exercise, intensity, age and other factors, between 20 and 50% of athletes suffer gastrointestinal symptoms, which have been shown to increase with exercise intensity [42]. Marathon, triathlete and ultra endurance athletes have been reported to have plasma LPS concentrations between 5 and 284 pg/mL and up to 93% of athletes reported digestive disturbances, which could be caused by the LPS-induced cytokine response [43].

In line with these results, the MFA was also able to pinpoint key miRNAs that regulate the immune and inflammatory response to endurance exercise such as the let-7 family, miR-21-5p, miR-16-5p and miR-26b-5p. For instance, the up-regulation of circulating miR-21-5p in the plasma of human endurance athletes has been reported [10–15], as well as in circulating peripheral blood mononuclear cells [44] and blood cells [45] upon exercise. Moreover, miR-16 had been shown to target specific genes involved in the Janus kinase-signal transducer and activator of transcription (STAT) pathway during exercise while modifying neutrophil immune function [46]. Although there is limited understanding of the role let-7 plays in the molecular pathways involved in the endurance exercise, it has been shown that let-7 might inhibit the pro-inflammatory IL6 expression indirectly through the NFκB pathway and can increase signaling pathway such as STAT [47]. Because the let-7 family has been detected in the horse liver, heart and muscles [48], further studies are needed to decipher whether let-7 might be biomarker of muscle damage, myocardial injury or endotoxemia during equine endurance events.

While this study provides novel insight into the dynamic changes that metabolome, transcriptome and miRNome undergo during endurance exercise, it also has some limitations. First, the overall experimental sample size was small (*n* = 10). Second, the metabolome profiles in the current work contained only 54 variables per spectrum, much fewer compared to the gene and miRNA expression profiling. Third, the presented regulatory network significantly depends on knowledge about the biochemical pathways structure and involved regulatory interactions described in the human literature. Fourth, working with large *p,* and small *n* was computationally challenging and rendered a more difficult interpretation of the relevant variables that provided more insight into the adaptation response of endurance exercise.

Nevertheless, our results suggest that this small sample size was appropriate because the true effects of endurance exercise being estimated were genuinely large enough to be reliably observed in the blood. Additionally, our results show that the limited number of metabolites measured was highly informative about the energy and redox status of the animals, as well as the protein catabolism and LPS-induced immune responses. Furthermore, we also demonstrated that although metabolites did not fit as neatly into the direct relationships of gene-transcript-protein [49] and that transcripts and metabolites may not be synchronized in time, the physiological relevance of metabolite variation can be examined through the relationships between the transcript levels of different enzymes and their up or downstream metabolites.

Finally, using a larger and independent cohort of endurance horses and MFA method, we were able to replicate the results observed in the small set of animals and confirm that the integrated analysis of different “omic” layers without a priori information provides more insights into the adaptive regulatory mechanisms to endurance exercise than any layer could by itself, highlighting the complementarity of an integrative approach. MFA method could be considered a useful approach to overcome computational issues when *p* > *n* and to display a low-dimensional projection of the data highlighting the main sources of variability.

## Conclusions

Our study highlights the potential of a systems-based approach for discovering the interactions between blood metabolome, transcriptome, and miRNome, which cannot be detected when analyzing single metabolites or transcripts. We were able to construct a regulatory network of 11 metabolites, 263 metabolic genes and 5 miRNAs whose expression was significantly altered at T1 (post- endurance competition) relative to T0 (baseline, pre- endurance competition). This network underlines essential adaptations necessary for homeostasis and performance during endurance exercise (i.e. energy sensing through FoxO and glucagon signaling pathways, as well as via activation of the mitochondrial oxidative phosphorylation system and AMPK system). Remarkably, the “omic” integration showed that lactate and glucose metabolites were enriched by genes that coded proteins likely involved in LPS-induced pro-inflammatory pathway, whereas glutamate and tyrosine were associated with genes that regulate the control and removal of ROS and RONS molecules.

In an independent cohort of 31 horses, multiple factor analysis confirmed the physiological adaptation to physical exertion through genes associated with LPS-induced immune responses and oxidative stress, as well as miR-21-5p, miR-16-5p, and let-7 family. Multiple factor analysis also shed light on key metabolomic and transcriptomic processes that occur in horses during endurance exercise, as well as potential transcriptomic markers at basal time for elimination during the endurance competition.

Integrating results from circulating plasma metabolites, transcriptome and miRNome derived from whole blood allowed us to (i) confirm that strenuous exercise in horses leads to coordinated transcriptome and metabolome reactions at a systemic level, increasing the metabolic rate, the production of reactive oxygen species, inflammation and compromising antioxidant defense system, (ii) identify potential candidate biomarkers at basal time that could predict the likelihood for failure to finish an endurance ride; and (iii) provide a basis for future studies to gain novel insights into the regulatory mechanisms that control physiological adaptations to endurance exercise.

## Methods

### Animals and samples

The data sets used for metabolome, transcriptome, and miRNome analysis have already been published by Le Moyec et al. [19] and Mach et al. [8].

Le Moyec et al. [19] evaluated the effects of long endurance exercise on the plasma metabolomic profiles, whereas Mach et al. [8] identified the whole blood miRNA-mRNA relationships specifically regulated by endurance exercise in the same horses. Altogether, we selected samples from a total of 41 pure-breed or half-breed Arabian horses (13 females and 28 geldings; mean ± SEM age: 9.7 ± 1.5) participating in a 160 km endurance competition. All horses were recruited on a volunteer basis in three different French competitions (see Additional file 1). For each of these 41 horses, the combined metabolome, transcriptome, and miRNome profiles were available. To ensure sample homogeneity, the participating horses were subjected to the same management practices throughout the endurance competition and passed the International Equestrian Federation (FEI)’s compulsory examination before the start. Animals were fed 2–3 h before the start of the endurance competition with ad libitum hay and 1 kg of concentrate pellets. During the endurance competition, all the animals underwent veterinary checks every 20 to 40 km, followed by recovery periods of 40 to 50 min (in accordance with the FEI rules of endurance riding). After each vet gate check, animals were provided with ad libitum water and hay and a small amount of concentrate pellets. The median winning speed over the entire endurance competition was 15.76 ± 1.04 km/h.

In our study, the 41 horses were divided into two non-overlapping sets: the experimental set and the validation set. The validation set came from an independent cohort of animals, to ensure that the observed effects were reproducible in a broader context. The experimental set included 10 horses sampled before the 160 km endurance competition (T0, baseline) and post- endurance competition (T1).

The validation set included 13 horses sampled solely at T0 and 18 other horses sampled solely at T1. Among the horses sampled at T0, three animals finished the endurance competition, while ten horses failed a vet gate check for lameness, metabolic disorders or tiredness (see Additional file 1). All horses sampled at T1 completed the endurance competition.

Blood samples for metabolome, transcriptome, and miRNome profiling were obtained from the jugular vein at rest (Basal, T0) and/or immediately after the end of the competition (T1). Pretreatment of the blood samples was carried out immediately after the collection because field conditions provided access to refrigeration and electrical power supply. Briefly, whole blood samples from each horse were collected in sodium fluoride and oxalate tubes for metabolome profiling in order to inhibit further glycolysis that may increase lactate levels after sampling. Whole blood drawn for plasma generation was refrigerated immediately at 4 °C to minimize the metabolic activity of cells and enzymes and kept the metabolite pattern almost stable. Clotting time at 4 °C was strictly controlled for all samples to avoid cell lyses that affect metabolome components. After clotting at 4 °C, the plasma was separated from the blood cells and subsequently transported to the lab at 4 °C and frozen at -80 °C (no more than 5 h later, in all cases). Additionally, blood samples were collected in dry tubes at the end of the endurance event for the biochemical analysis. After clotting at room temperature, the tubes were centrifuged and the harvested serum was stored at 4 °C until analysis (no more than 48 later, in all cases). PAXgene Blood RNA tubes (Qiagen, Hilden, Germany) were used for transcriptome and miRNome profiling. They were kept at room temperature for no more than 5 h in all cases, and stored at -80 °C until analysis.

### ^1^H NMR data acquisition and statistical analysis

The ^1^H NMR spectra were acquired with a Bruker® Advance II spectrometer (Bruker BioSpin, Wissembourg, France) operating at 500 MHz and using a standard water-suppressed 1D spectrum (NOESY1D) sequence for the preservation of lipid signal intensities.

The methods for sample preparation, data acquisition, data quality control, spectroscopic data-pre-processing, and data pre-processing including peak alignment, scaling and normalization are broadly explained elsewhere [19]. In contrast to Le Moyec et al. [19], where *in silico* metabolite identification and statistics were done by the MATLAB commercial program, we used different open-source R packages, allowing automated qualitative and quantitative metabolite characterization, calculation of spatial significance and, importantly, metabolite pathway analysis.

Peaks with just one non-zero intensity (single mass events) were removed from the normalized matrix. Additionally, a metabolite peak was considered to be detectable only if it was expressed in at least 50% of the experimental samples. The metabolite identification was then performed by using structure message of metabolites acquired from other available biochemical databases, such as human metabolome database (HMD), http://www.hmdb.ca; KEGG, http://www.genome.jp/kegg/; METLIN, http://metlin.scripps.edu/; Chemical Entities of Biological Interest (http://www.ebi.ac.uk/Databases/); and Lipidmaps (http://www.lipidmaps.org/). Metabolite assignment of each peak was considered when chemical shifts of peaks in the samples were the same as in the publicly available reference databases (with a shift tolerance level of ± 0.005 ppm), in order to counter-act the effects of measurements and pre-processing variability introduced by factors such as pH values and solvents. A manual curation for identified compounds was done by an expert in horse metabolomics [19, 20]. Afterwards, the relative abundance of each metabolite was calculated as the area of the peak [50].

Exploratory analysis of the metabolites signals were first performed by PCA, which displays the internal structure of data sets in an unbiased manner and reduces data dimensionality through linear combinations of the original variables. A PCA score plot was used to reveal the presence of outliers outside the 95% significance region of ellipse (i.e., strong outliers). Interclass PCA of plasma metabolites with pre and post- endurance competition samples as an instrumental variable was also performed, based on a Monte Carlo test with 999 replicates. Unsupervised and supervised methods were performed with a toolbox to explore NMR metabolomic data sets in the R environment [51]. Then, we performed supervised projections to latent structures-Discriminant Analysis (PLS-DA) and the OPLS analysis, which integrates an orthogonal signal correction (OSC) to identify and characterize metabolic changes induced by endurance exercise. The OSC-correction approach was conducted using DeviumWeb R package; (https://github.com/dgrapov/DeviumWeb) [52].

By extracting variation from its computed partial least squares (PLS) components that is uncorrelated (orthogonal) to the responses, OPLS produces a more interpretable regression model compared to PLS [53]. Loading plots combining the reliability and correlation from the OPLS models were used to identify differential metabolites among the pre and post- endurance competition samples. In the loading plots, signals with a positive direction corresponded to metabolites that were present at high concentrations at T1. The negative direction indicated a negative direction of metabolite values at T1. A high loading score means that the metabolite in question has an excellent ability to separate pre and post-endurance competition samples. Importantly, only metabolites with loadings plots different from zero along any OPLS principal component axis were considered to have a contribution to the model. These metabolites were considered to be biological relevant and suitable for downstream analysis.

The 10-fold within model cross validation and permutation Monte Carlo testing (*n* = 1,000) were applied for internal validation of the Q^2^, R^2^ and the RMSEP values, which represent the predictability of the model, the explained variance and the error, respectively. The firmness of the model was evaluated with R^2^ outcomes and the precision of the prediction (Q^2^).

Complementary to the multivariate statistical methods to identify metabolites that were statistically different between T0 and T1 samples, analysis of variance (ANOVA) was performed to delineate whether there was a significant difference between the average values of metabolites normalized intensities between pre and post- endurance competition samples. Because of the multiple testing issues, Bonferroni corrected *P*-values were calculated. A significance level of corrected *p* < 0.05 was accepted.

This data is available at the NIH Common Fund’s Data Repository and Coordinating Center (supported by NIH grant, U01-DK097430) website, http://www.metabolomicsworkbench.org), where it has been assigned a Metabolomics Workbench Project ID: (UrqK1489). The data is directly accessible at: http://dev.metabolomicsworkbench.org:22222/data/DRCCMetadata.php?Mode=Study&DataMode=AllData&StudyID=ST000503&StudyType=NMR&ResultType=2&access=UrqK1489#DataTabs

### Metabolic pathway construction

Metabolic pathways were constructed according to pathway analysis on Metpa (freely available at http://metpa.metabolomics.ca). Metpa high-level functional analysis is organism specific. Because the metabolite pathways for *Equus caballus* do not currently exist, the enrichment analysis was performed using *Homo sapiens* metabolite sets.

The MetPA’s pathway’s topological analysis is based on the centrality measures of a metabolite in a given metabolic network [54]. Centrality is a local quantitative measure of the position of a node relative to the other nodes, and is often used to estimate a node’s relative importance or role in network organization [54]. Since metabolic networks are directed graphs, MetPA uses relative betweenness centrality and out degree centrality measures to calculate compound importance. The impact of a pathway is calculated as the sum of the importance measures of the matched metabolites normalized by the sum of the importance measures of all metabolites in each pathway [54].

### RNA isolation, microarray experiments and mRNA and miRNA statistical analysis

Transcriptome and miRNome profiling were performed using Agilent microarrays as described earlier [8]. All the transcriptome and miRNome pre-processing, normalization and statistical analysis steps were carried out as described elsewhere [8]. In contrast to the Mach et al. [8] study, the *p*-values were corrected for multiple testing using the Benjamini and Hochberg method with a threshold of adjusted *p* < 0.05, as a compromise between the unadjusted analysis and the Bonferroni procedures.

The data sets supporting the conclusions of this article are available in the Gene Expression Omnibus (GEO) repository, [GSE73104; (http://www.ncbi.nlm.nih.gov/geo/query/acc.cgi?acc=GSE73104].

### Regulatory network of metabolites, metabolic genes and miRNAs: enrichment analysis

The underlying assumption behind the enrichment analysis is that by combining the evidence based on changes in both gene expression and metabolite concentrations, one is more likely to pinpoint the metabolites and pathways involved in the underlying biological processes during endurance exercise. The power of this approach is that such models can provide non-intuitive metabolic and physiological hypothesis [55].

First of all, we converted the equine Ensembl gene IDs to their orthologous associated *Homo sapiens* gene IDs through the Biomart retrieval tool (Ensembl release 83; http://www.ensembl.org/biomart/).

Then after, for each metabolite with a loading different from zero along any OPLS principal component axis, we identified all the metabolic pathways that the given metabolite participates in through the KEGGREST package in R (*Homo sapiens,* [hsa] organism). We retrieved the list of genes that encode proteins that participate in the reaction steps around the given metabolic pathway (using the KeggGet function from the KEGGREST package in R). The enzyme-encoding genes whose protein products participate in the metabolic reaction of a given metabolite are referred to here as metabolic genes. We considered metabolic genes as targets of a specific metabolite when they were operating in the metabolic pathways that the metabolite participates in. Each metabolite may be associated with multiple metabolic pathways, and each metabolic gene may be involved in several metabolic pathways.

To investigate the metabolites, transcripts and pathways with more biological relevance during the endurance exercise, we used two complementary statistical approaches. For each of the metabolites presenting at least one metabolic pathway with one coding metabolic gene, we performed: (i) the hypergeometric test for enrichment in target metabolic genes correlated with the metabolite; and (ii) the generalization of the hypergeometric test. Both methods were implemented on the experimental set of ten animals. For the hypergeometric test, we performed a pair-wise Pearson correlation analysis of the expression levels of DEGs and the expression levels of metabolites presenting at least one target metabolic gene. Subsequently, we subtracted the set of correlated metabolites-DEGs with r | > 0.5|; with a *p* < 0.05. We next looked at whether the metabolic target genes were over-represented when compared with the other genes in the transcriptome. Benjamini and Hochberg correction [56] for multiple testing was applied to the *p*-values obtained (false discovery rate (FDR) < 0.10).

Moreover, in order to address the hypergeometric test’s lack of power when the numbers of metabolic target genes for a given metabolite was very small, we also implemented a variant of the hypergeometric test. In this variant, we tested for enrichment in metabolic target genes by selecting DEGs regardless of the sign and value of their correlation with the metabolite. When considering a given metabolite with at least one metabolic target gene, we looked at whether their metabolic target genes presented smaller *p*-values than the other genes in the transcriptome using a one-sided Wilcoxon rank sum test (implemented with the “wilcox.test” function in R). The FDR was determined to correct for multiple testing. Lastly, only metabolites that were significant either in the hypergeometric test or its generalization after correction for multiple testing (FDR < 0.1) were analyzed further. The FDR was set to 0.1 in order to retain as many biological functions as possible.

### Identification of miRNAs involved in the regulation of the identified metabolite-metabolic target genes regulatory network

The multiMiR package in R was used to identify potential miRNAs regulating the metabolic target genes included in the regulatory network [57]. For each miRNA expressed in our data set, we assembled a comprehensive list of all experimentally validated target genes using the multiMiR package in R. multiMiR is a comprehensive collection of predicted and validated miRNA–target interactions and their associations with diseases and drugs [58]. It contains human and murine data from 14 external databases that are categorized into three components, including three validated miRNA–target databases (miRecords, miRTarBase and TarBase), eight predicted miRNA–target databases (DIANA-microT, ElMMo, MicroCosm, miRanda, miRDB, PicTar, PITA and TargetScan), and three disease- or drug-related miRNA databases (miR2Disease, Pharmaco-miR and PhenomiR) [58]. Subsequently, we subtracted the set of miRNAs presenting at least one metabolic target gene within our regulatory network.

### Regulatory impact factors analysis to unravel the transcription regulation within the regulatory network

The RIF metric [28, 29] was used to identify transcription factors and miRNAs regulators within the set of DEGs. RIF method contrasts two conditions (e.g., case vs. control) and provides a metric to each regulator considering the change in co-expression between the regulator and DEGs. In this study, we focused on genes whose expression was significantly altered at T1 relative to T0, with an adjusted *p* < 0.05, and we used them as putative regulators for all transcription factors reported by Vaquerizas et al. [59] and the 362 miRNAs expressed in the custom microarray (Additional file 14). The method estimated two RIF alternative measures (RIF1 and RIF2). RIF1 provides information of those regulators that are consistently more differentially co-expressed with the highly abundant and the highly differentially expressed genes. RIF2 scores each regulator considering the most altered ability to act as predictors of the abundance of DEGs.

### Multiple factor analysis in an independent cohort of 31 horses

The MFA [60] was applied as an exploratory analysis of the metabolome, transcriptome, and miRNome using FactoMineR [61] R package. MFA was applied in our independent cohort of animals (*n* = 31) to model complex biological interaction in a holistic manner and identify potential biomarkers with a functional readout of the cellular state. MFA is an extension of PCA tailored to handle multiple data tables that measure sets of variables collected on the same individuals. MFA proceeds in three steps: first, it computes a PCA of each subset of variables and ‘normalizes’ each data table by dividing all its elements by the first singular value obtained from its PCA. In other words, it weights each data table to account from different variances among groups. Second, all the normalized data tables are aggregated into a grand data table that is analyzed via a (non-normalized) PCA that gives a set of factor scores for the observations and loadings for the variables. Third, in order to identify the contribution of each data set to the total variance, that is, to what extent each data set deviates or agrees with what the majority of data sets support, MFA projects a superimposed representation of individuals with each group of data and its barycenter. MFA also provides a measurement of similarity between the geometrical representations derived from each group of variables though a RV coefficient, which is a multivariate generalization of the Pearson correlation coefficient. For each pair of “omic” data sets, the RV-coefficient is calculated as the total co-inertia (sum of eigenvalues of co-inertia, i.e. sum of eigenvalues of the product of two cross product matrices) divided by the square root of the product of the squared total inertia (sum of the eigenvalues) from the individual analysis. As the co-structure between two data sets increases, the RV score move towards to 1. A zero RV score indicates no similarity.

## Additional files

Additional file 1: The horses’ morphological and physiological parameters. (XLS 49 kb)
Additional file 2: Biochemical parameters obtained from 41 blood samples collected after the 160 km endurance competition. Samples were obtained for ten horses from the experimental set and 31 horses from the validation set. (XLS 34 kb)
Additional file 3:
^1^H NMR metabolites present in the plasma of horses following endurance exercise. (XLS 33 kb)
Additional file 4: Figure S1. Differential metabolite expression profiles in plasma. (A) PCA of metabolites in plasma when comparing T1 with T0. The first axis accounted for 27.91% of the total variance, and the first two components accounted for 79.79% of the total variance (*p* < 0.05); (B) PLS-DA plot scores. Discrimination in the first and second component indicates metabolic differences between pre and post- endurance competition samples. In all cases, individual horses are represented as purple dots (for T0) and green dots (for T1); (C) OPLS-loading plot represents the enhanced metabolites in plasma in pre- and post- endurance competition samples. A positive loading score indicated there was a relatively greater concentration of metabolite present in post- endurance competition samples and a negative loading score indicated a relatively lower concentration, with respect to pre- endurance competition samples. (TIF 21383 kb)
Additional file 5: Figure S2. Unsupervised analysis on metabolite expression profiles in plasma. (A) Correlation network with a threshold of 0.8 between the metabolites; (B) Heatmap image of the correlations; (C) Boxplot of the expression of the main metabolites in plasma. Individual horses are represented as purple color (for T0) and green color (for T1). *, **, *** denote statistical significance at the 0.10, 0.05 and 0.001 level respectively, after multiple testing correction using the Bonferroni method. (TIF 21557 kb)
Additional file 6: Figure S3. A functional map of the metabolites affected by the endurance exercise. The metabolic pathways were constructed according to pathway analysis on Metpa. The size of the node corresponds to the statistical significance of the enrichment term together with the impact. (TIF 13708 kb)
Additional file 7: Differentially expressed genes in 10 animals following endurance exercise (a 160 km endurance competition). (XLS 1568 kb)
Additional file 8: Differentially expressed miRNAs in 10 animals following endurance exercise (a 160 km endurance competition). (XLSX 65 kb)
Additional file 9: Figure S4. Overview of the data analysis. Step 1: a linear model analysis of DEGs and DEmiRNAs and a supervised analysis of metabolites. Step 2: determination of the corresponding gene ontology (GO) terms, Kyoto encyclopedia of genes and genomes (KEGG) pathways and TFs and miRNAs regulating the DEGs. Step 3: selection of metabolic target genes involved in the pathways that metabolites participate in. Step 4: generation of the correlation matrix for DEGs and metabolites (in the hypergeometric test only). Step 5: the enrichment test (using the hypergeometric test or its generalization) used to select candidate-enriched metabolites. Step 6: the functional regulatory network analysis (TFs and miRNAs); Step 7: validation of the regulatory network, using an independent cohort and multiple factor analysis. (TIF 8893 kb)
Additional file 10: Enriched metabolites. (XLS 40 kb)
Additional file 11: RIF scores according to RIF1 or RIF2 for transcription factors and miRNAs. Only molecules with RIF *z*-scores < −1.40 or > 1.40 are described. (XLS 52 kb)
Additional file 12: Factor scores of multiple factor analysis for the observations on the axis 1 and 2 in the validation set at T0. (XLS 124 kb)
Additional file 13: Factor scores of multiple factor analysis for the observations on the axis 1 and 2 in the validation set at T1. (XLS 316 kb)
Additional file 14: Expressed miRNAs. (XLSX 159 kb)

## Abbreviations

^1^H NMR: Proton nuclear magnetic resonance
ACAB: Acetyl-CoA carboxylase
ACSS: Acyl-CoA synthetase short-chain family
AHCTF1: AT-hook containing transcription factor 1
AMPK: Activated protein kinase
ANOVA: Analysis of variance
ATP5: ATP Synthase, H+ Transporting, Mitochondrial F1 Complex
BCL6: B-cell lymphoma 6 protein
COPR5: Histone binding protein 5
COX: Cytochrome oxidase subunits
CREBBP: CREB Binding Protein
DEGs: Differentially expressed genes
DEmiRNAs: miRNAs differentially expressed
EP300: E1A binding protein P3
F-1: 6-BP, Fructose-1,6- bisphosphate
F-6-P: Fructose 6-phosphate
FDR: False discovery rate
FEI: International Equestrian Federation
FoxO: Forkhead box protein O
G-6-P: Glucose 6-phosphate
GEO: Gene expression Omnibus
GO: Gene ontology
GSH: Reduced glutathione
GSSG: Oxidized glutathione
GSTK1: GST-kappa 1
GTS: Glutathione S-transferase
HIF1A: Hypoxia-inducible factor A
HMD: Human metabolome database
HPA: Hypothalamus-pituitary-adrenal
HSF1: Stress-induced heat-shock factor 1
IFN: Interferon-alpha
IFNGR-1: Interferon-gamma receptor, ligand-binding chain (alpha)
IL: Interleukin
IRAK2: Interleukin-1 receptor-associated kinase-like 2
KEGG: Kyoto encyclopedia of genes and genomes
LPS: Lipopolysaccharides
MAF: Multiple factor analysis
MGST: Microsomal GST-kappa 1
MRPL22: Mitochondrial ribosomal protein L22
MRPL35: Mitochondrial ribosomal protein L35
ND: NADH: ubiquinone oxidoreductase core subunits
NDUFB5: NADH dehydrogenase (ubiquinone) 1 beta sub-complex 5
NFkβ: Nuclear factor kappa beta
NOESY1D: Standard water-suppressed 1D spectrum
OPLS: Orthogonal projections to latent structures
OSC: Orthogonal signal correction
OXPHOS: Oxidative phosphorylation
PC: Principal component 1
PCA: Principle component analysis
PEP: Phosphoenolpyruvate
PLS: Partial least squares
PLS-DA: Latent structures-discriminant analysis
POLR2I: RNA polymerase 21-kDA subunit
R-5-P: Ribose-5-phosphate.
RBL2: Retinoblastoma-Like 2
RIF: Regulatory impact factor
RMSEP: Root mean square error of prediction
RONS: Nitrogen oxide species
ROS: Reactive oxygen species
SAA: Serum amyloid protein
SAT2: N1-acetyltransferase family member 2
SIRT1: Sirtuin 1
SSBP1: Mitochondrial single-stranded DNA-binding protein
STAT3: Activator of transcription 3
T0: Baseline, pre-endurance competition
T1: Post- endurance competition
TCA: Tricarboxylic acid
TF: Transcription factor
TLR4: Toll-like receptor 4
TNFAIP3: TNF alpha induced protein 3
TOR3A: Torsin 3A
TRIM65: Tripartite motif containing 65
UBE3C: E3 ligases
VAMP: Vesicle-associated membrane protein 5
