# Supplementary figures and images for: Understanding the response to endurance exercise using a systems biology approach: combining blood metabolomics, transcriptomics and miRNomics in horses
Source: BMC Genomics. 2017 Feb 17;18:187. doi: 10.1186/s12864-017-3571-3 (PMC5316211; doi:10.1186/s12864-017-3571-3)

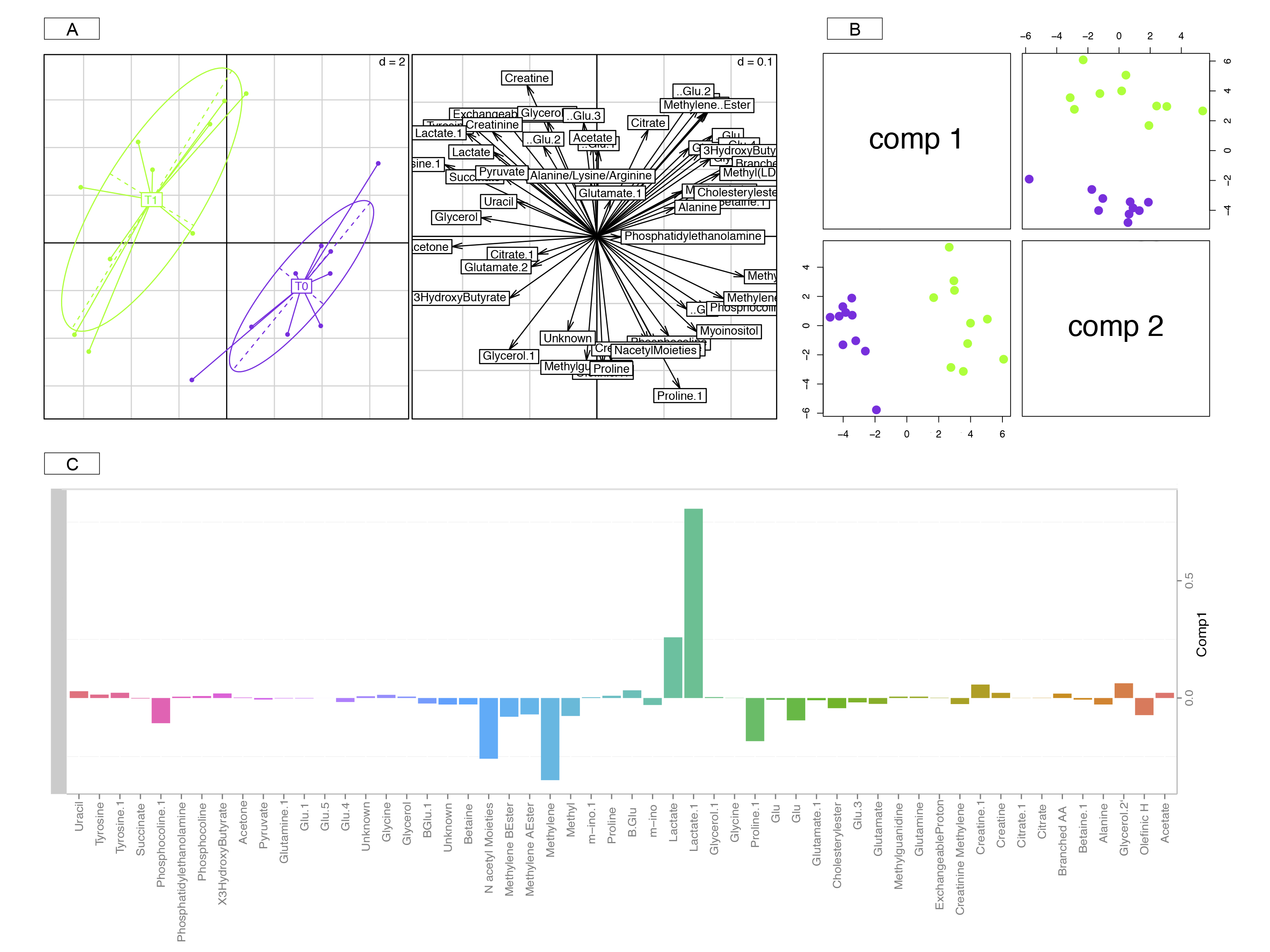

Supplement: Additional file 4: Figure S1. — Differential metabolite expression profiles in plasma. (A) PCA of metabolites in plasma when comparing T1 with T0. The first axis accounted for 27.91% of the total variance, and the first two components accounted for 79.79% of the total variance (p < 0.05); (B) PLS-DA plot scores. Discrimination in the first and second component indicates metabolic differences between pre and post- endurance competition samples. In all cases, individual horses are represented as purple dots (for T0) and green dots (for T1); (C) OPLS-loading plot represents the enhanced metabolites in plasma in pre- and post- endurance competition samples. A positive loading score indicated there was a relatively greater concentration of metabolite present in post- endurance competition samples and a negative loading score indicated a relatively lower concentration, with respect to pre- endurance competition samples. (TIF 21383 kb) [file 12864_2017_3571_MOESM4_ESM.tif]

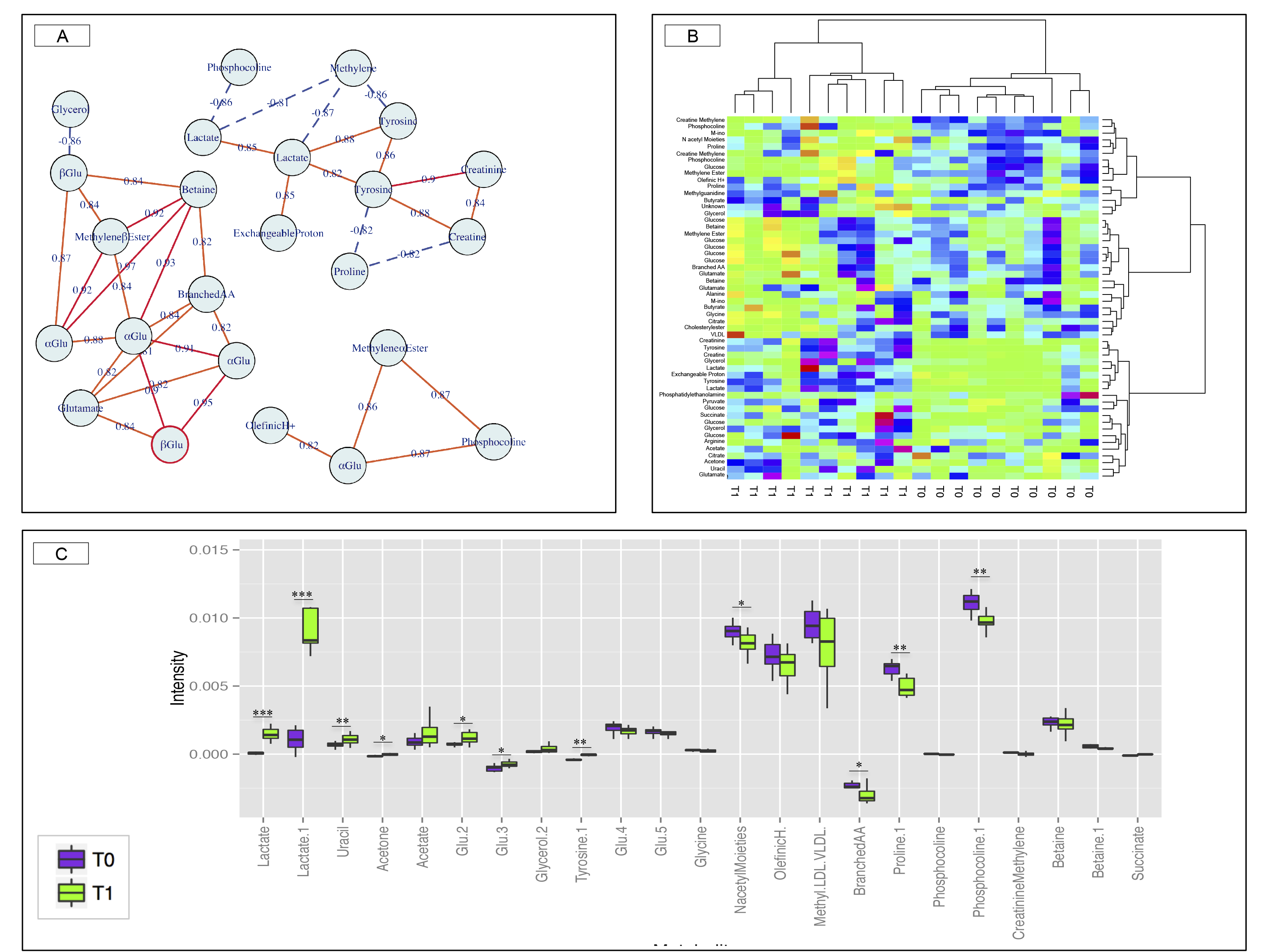

Supplement: Additional file 5: Figure S2. — Unsupervised analysis on metabolite expression profiles in plasma. (A) Correlation network with a threshold of 0.8 between the metabolites; (B) Heatmap image of the correlations; (C) Boxplot of the expression of the main metabolites in plasma. Individual horses are represented as purple color (for T0) and green color (for T1). *, **, *** denote statistical significance at the 0.10, 0.05 and 0.001 level respectively, after multiple testing correction using the Bonferroni method. (TIF 21557 kb) [file 12864_2017_3571_MOESM5_ESM.tif]

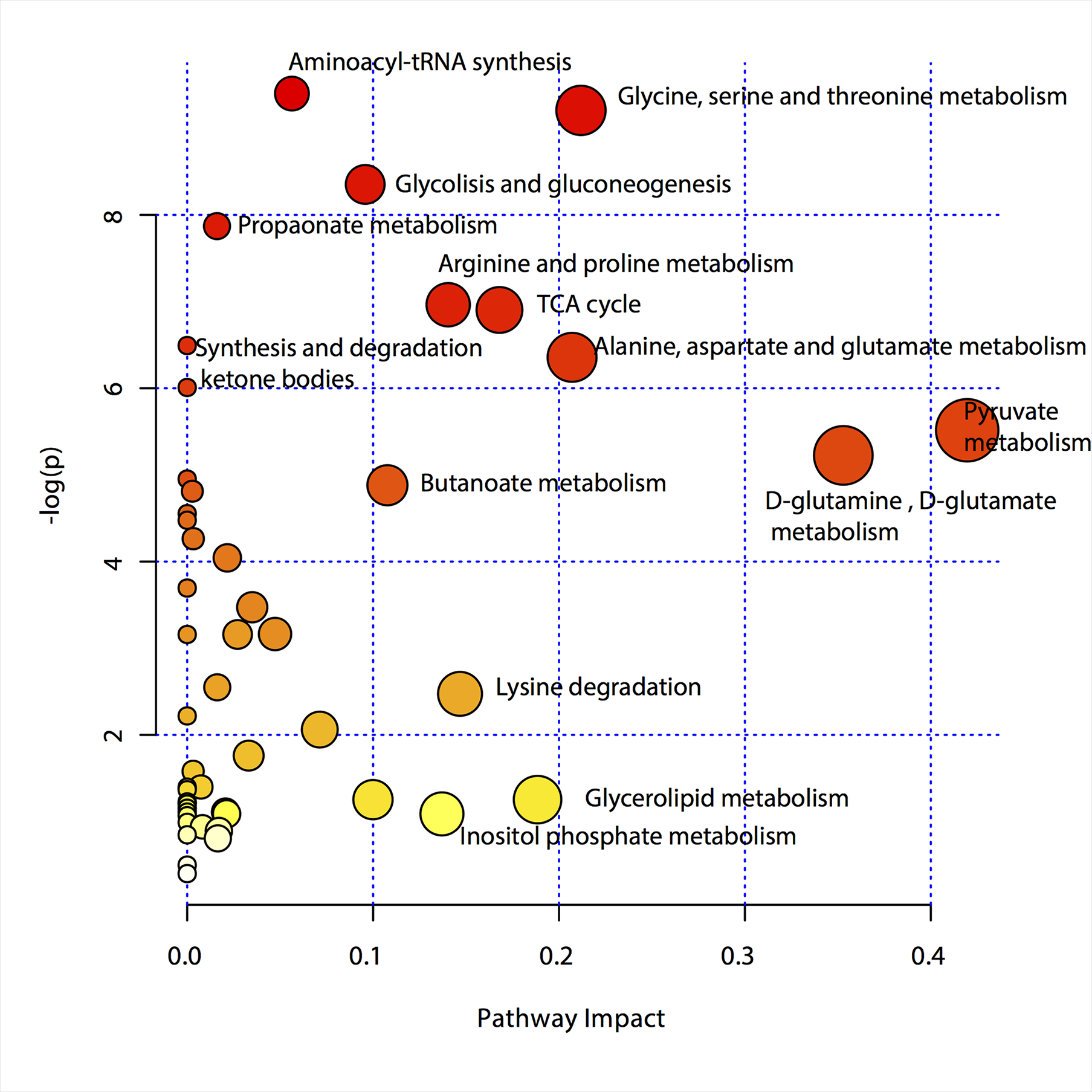

Supplement: Additional file 6: Figure S3. — A functional map of the metabolites affected by the endurance exercise. The metabolic pathways were constructed according to pathway analysis on Metpa. The size of the node corresponds to the statistical significance of the enrichment term together with the impact. (TIF 13708 kb) [file 12864_2017_3571_MOESM6_ESM.tif]

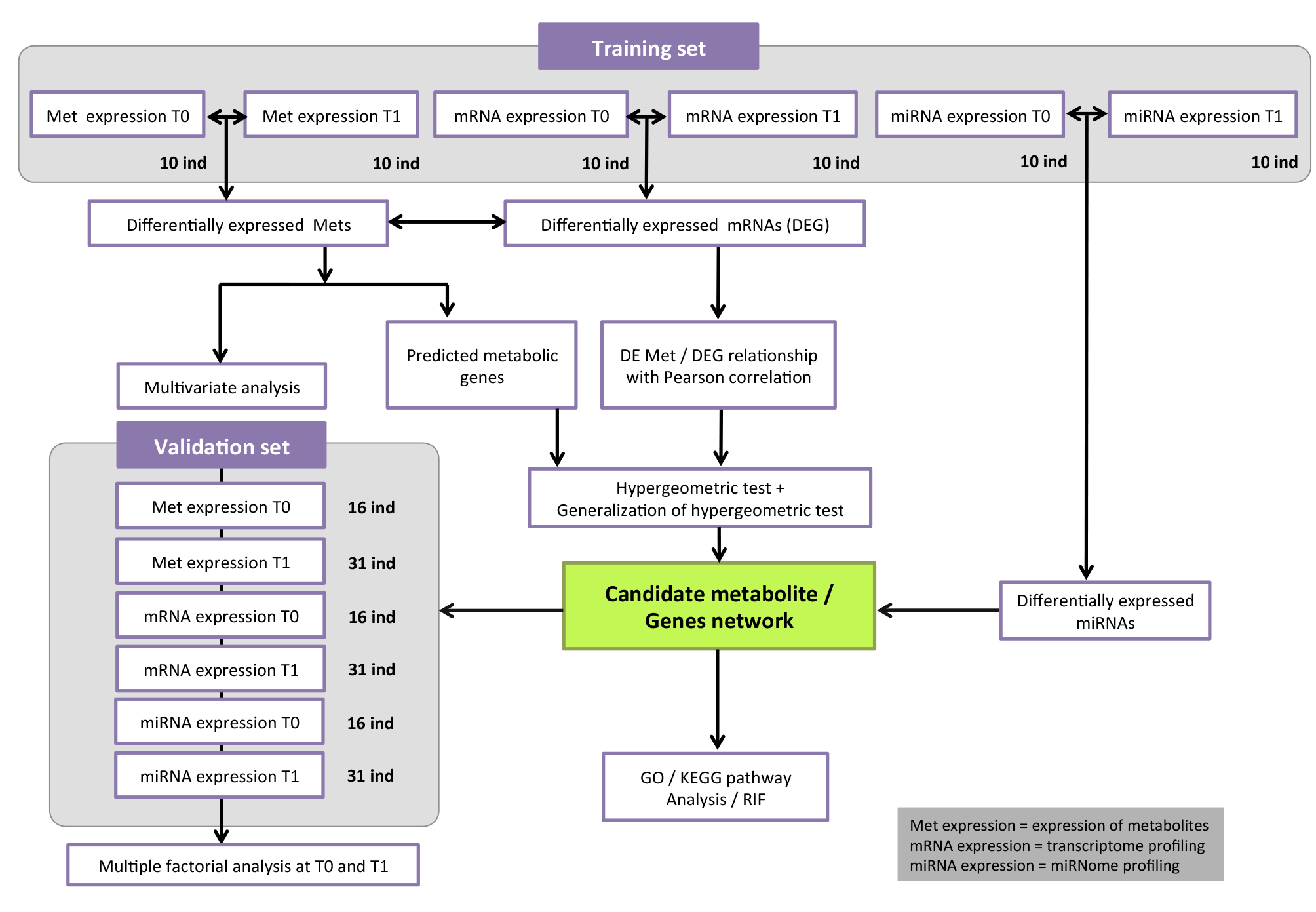

Supplement: Additional file 9: Figure S4. — Overview of the data analysis. Step 1: a linear model analysis of DEGs and DEmiRNAs and a supervised analysis of metabolites. Step 2: determination of the corresponding gene ontology (GO) terms, Kyoto encyclopedia of genes and genomes (KEGG) pathways and TFs and miRNAs regulating the DEGs. Step 3: selection of metabolic target genes involved in the pathways that metabolites participate in. Step 4: generation of the correlation matrix for DEGs and metabolites (in the hypergeometric test only). Step 5: the enrichment test (using the hypergeometric test or its generalization) used to select candidate-enriched metabolites. Step 6: the functional regulatory network analysis (TFs and miRNAs); Step 7: validation of the regulatory network, using an independent cohort and multiple factor analysis. (TIF 8893 kb) [file 12864_2017_3571_MOESM9_ESM.tif]
